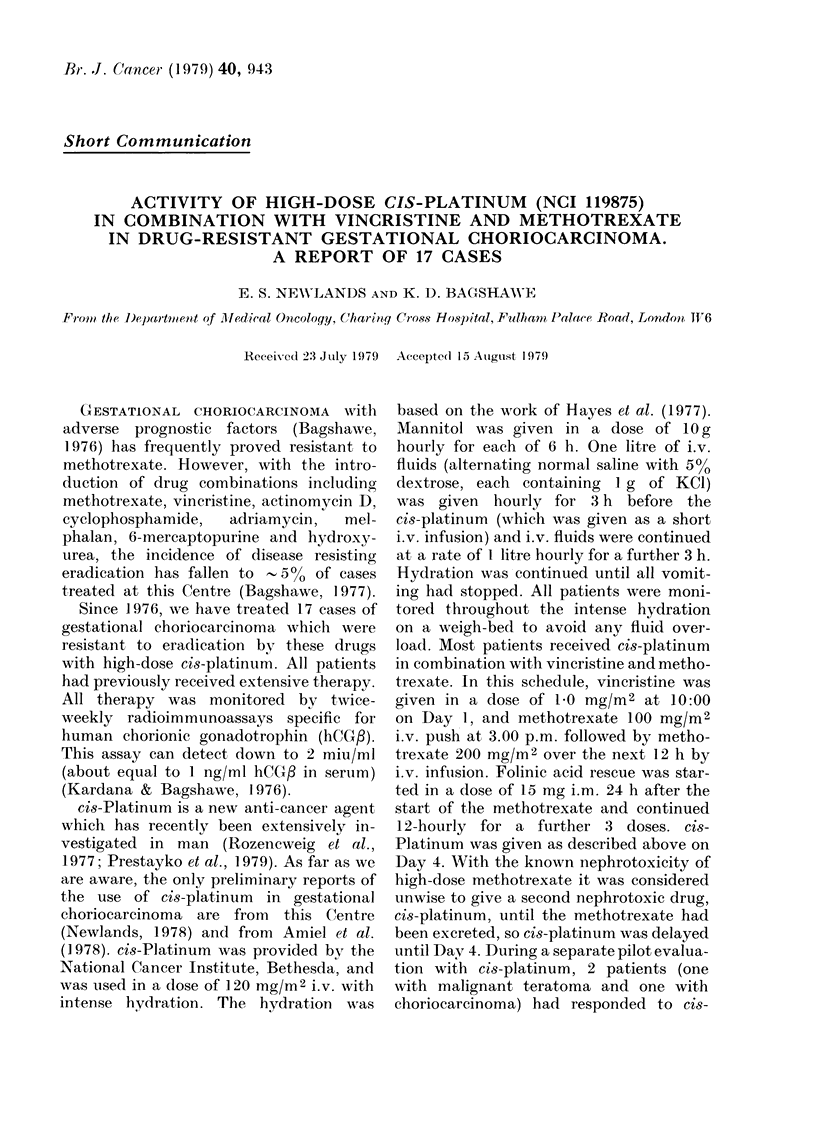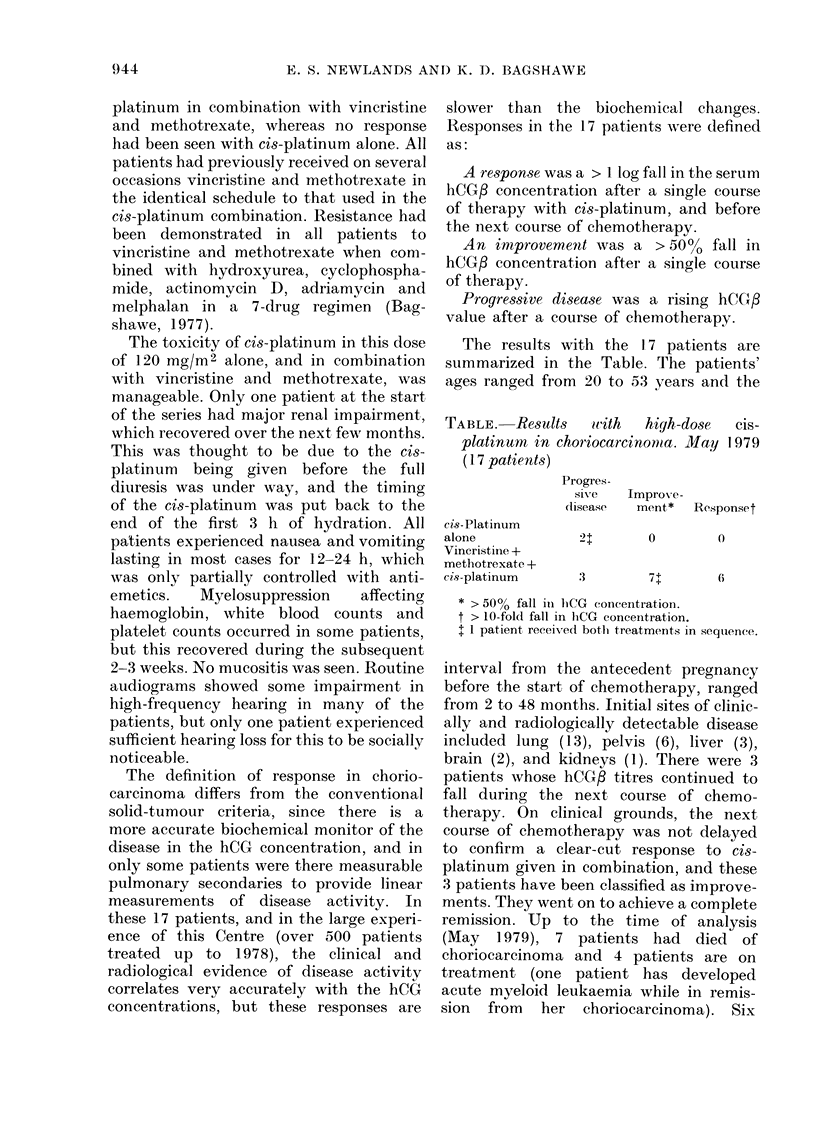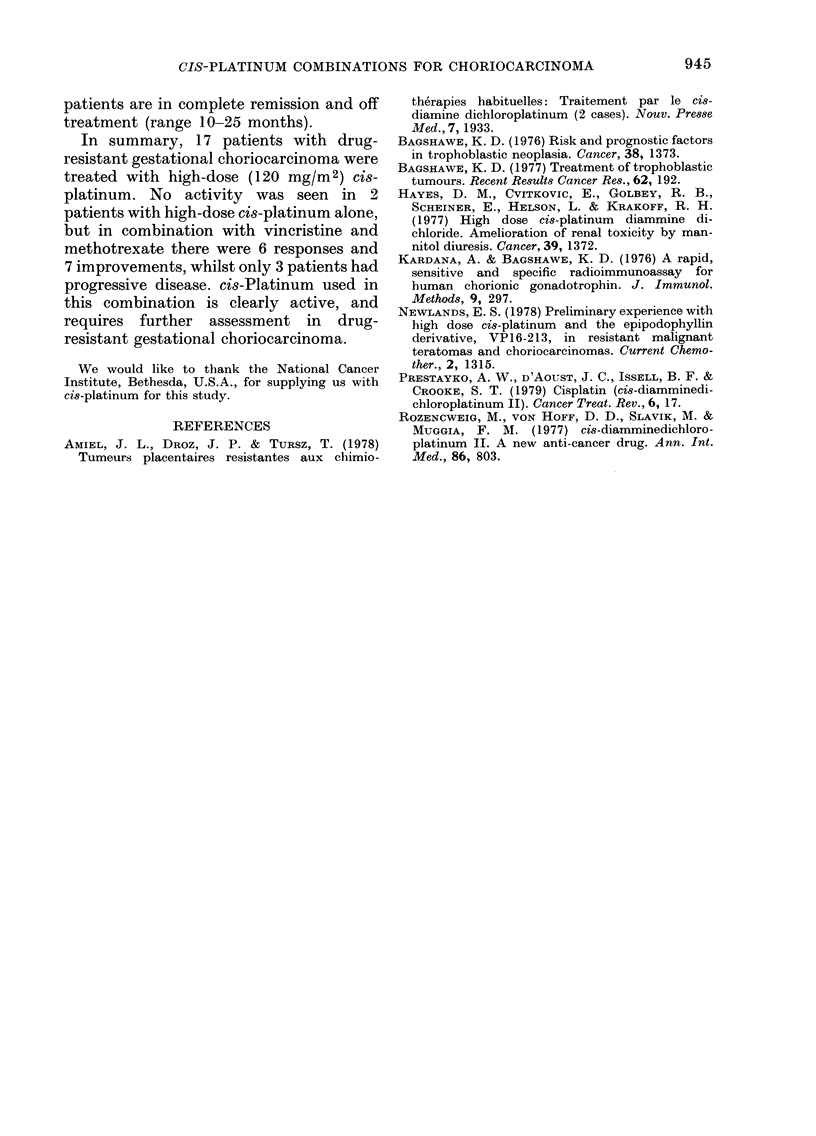# Activity of high-dose cis-platinum (NCI 119875) in combination with vincristine and methotrexate in drug-resistant gestational choriocarcinoma. A report of 17 cases.

**DOI:** 10.1038/bjc.1979.289

**Published:** 1979-12

**Authors:** E. S. Newlands, K. D. Bagshawe


*Br. J. Cancer* (1979) **40**, 943

### *Short Communication*

### ACTIVITY OF HIGH-DOSE *CIS*-PLATINUM (NCI 119875) IN COMBINATION WITH VINCRISTINE AND METHOTREXATE IN DRUG-RESISTANT GESTATIONAL CHORIOCARCINOMA. A REPORT OF 17 CASES

E. S. NEWLANDS AND K. D. BAGSHAWE

*From the Department of Medical Oncology, Charing Cross Hospital, Fulham Palace Road, London W6*

Received 23 July 1979 Accepted 15 August 1979

GESTATIONAL CHORIOCARCINOMA with adverse prognostic factors (Bagshawe, 1976) has frequently proved resistant to methotrexate. However, with the introduction of drug combinations including methotrexate, vincristine, actinomycin D, cyclophosphamide, adriamycin, melphalan, 6-mercaptopurine and hydroxyurea, the incidence of disease resisting eradication has fallen to ~5% of cases treated at this Centre (Bagshawe, 1977).

Since 1976, we have treated 17 cases of gestational choriocarcinoma which were resistant to eradication by these drugs with high-dose *cis*-platinum. All patients had previously received extensive therapy. All therapy was monitored by twice-weekly radioimmunoassays specific for human chorionic gonadotrophin (hCGβ). This assay can detect down to 2 miu/ml (about equal to 1 ng/ml hCGβ in serum) (Kardana & Bagshawe, 1976).

*cis*-Platinum is a new anti-cancer agent which has recently been extensively investigated in man (Rozencweig *et al.*, 1977; Prestayko *et al.*, 1979). As far as we are aware, the only preliminary reports of the use of *cis*-platinum in gestational choriocarcinoma are from this Centre (Newlands, 1978) and from Amiel *et al.* (1978). *cis*-Platinum was provided by the National Cancer Institute, Bethesda, and was used in a dose of 120 mg/m$^2$ i.v. with intense hydration. The hydration was based on the work of Hayes *et al.* (1977). Mannitol was given in a dose of 10 g hourly for each of 6 h. One litre of i.v. fluids (alternating normal saline with 5% dextrose, each containing 1 g of KCl) was given hourly for 3 h before the *cis*-platinum (which was given as a short i.v. infusion) and i.v. fluids were continued at a rate of 1 litre hourly for a further 3 h. Hydration was continued until all vomiting had stopped. All patients were monitored throughout the intense hydration on a weigh-bed to avoid any fluid overload. Most patients received *cis*-platinum in combination with vincristine and methotrexate. In this schedule, vincristine was given in a dose of 1·0 mg/m$^2$ at 10:00 on Day 1, and methotrexate 100 mg/m$^2$ i.v. push at 3.00 p.m. followed by methotrexate 200 mg/m$^2$ over the next 12 h by i.v. infusion. Folinic acid rescue was started in a dose of 15 mg i.m. 24 h after the start of the methotrexate and continued 12-hourly for a further 3 doses. *cis*-Platinum was given as described above on Day 4. With the known nephrotoxicity of high-dose methotrexate it was considered unwise to give a second nephrotoxic drug, *cis*-platinum, until the methotrexate had been excreted, so *cis*-platinum was delayed until Day 4. During a separate pilot evaluation with *cis*-platinum, 2 patients (one with malignant teratoma and one with choriocarcinoma) had responded to *cis*-

platinum in combination with vincristine and methotrexate, whereas no response had been seen with *cis*-platinum alone. All patients had previously received on several occasions vincristine and methotrexate in the identical schedule to that used in the *cis*-platinum combination. Resistance had been demonstrated in all patients to vincristine and methotrexate when combined with hydroxyurea, cyclophosphamide, actinomycin D, adriamycin and melphalan in a 7-drug regimen (Bagshawe, 1977).

The toxicity of *cis*-platinum in this dose of 120 $mg/m^2$ alone, and in combination with vincristine and methotrexate, was manageable. Only one patient at the start of the series had major renal impairment, which recovered over the next few months. This was thought to be due to the *cis*-platinum being given before the full diuresis was under way, and the timing of the *cis*-platinum was put back to the end of the first 3 h of hydration. All patients experienced nausea and vomiting lasting in most cases for 12–24 h, which was only partially controlled with antiemetics. Myelosuppression affecting haemoglobin, white blood counts and platelet counts occurred in some patients, but this recovered during the subsequent 2–3 weeks. No mucositis was seen. Routine audiograms showed some impairment in high-frequency hearing in many of the patients, but only one patient experienced sufficient hearing loss for this to be socially noticeable.

The definition of response in choriocarcinoma differs from the conventional solid-tumour criteria, since there is a more accurate biochemical monitor of the disease in the hCG concentration, and in only some patients were there measurable pulmonary secondaries to provide linear measurements of disease activity. In these 17 patients, and in the large experience of this Centre (over 500 patients treated up to 1978), the clinical and radiological evidence of disease activity correlates very accurately with the hCG concentrations, but these responses are slower than the biochemical changes. Responses in the 17 patients were defined as:

*A response* was a > 1 log fall in the serum hCG$\beta$ concentration after a single course of therapy with *cis*-platinum, and before the next course of chemotherapy.

*An improvement* was a >50% fall in hCG$\beta$ concentration after a single course of therapy.

*Progressive disease* was a rising hCG$\beta$ value after a course of chemotherapy.

The results with the 17 patients are summarized in the Table. The patients' ages ranged from 20 to 53 years and the interval from the antecedent pregnancy before the start of chemotherapy, ranged from 2 to 48 months. Initial sites of clinically and radiologically detectable disease included lung (13), pelvis (6), liver (3), brain (2), and kidneys (1). There were 3 patients whose hCG$\beta$ titres continued to fall during the next course of chemotherapy. On clinical grounds, the next course of chemotherapy was not delayed to confirm a clear-cut response to *cis*-platinum given in combination, and these 3 patients have been classified as improvements. They went on to achieve a complete remission. Up to the time of analysis (May 1979), 7 patients had died of choriocarcinoma and 4 patients are on treatment (one patient has developed acute myeloid leukaemia while in remission from her choriocarcinoma). Six

TABLE.—*Results with high-dose cis-platinum in choriocarcinoma. May* 1979 (17 *patients*)

| | Progressive disease | Improvement* | Response† |
|---|---|---|---|
| *cis*-Platinum alone | 2‡ | 0 | 0 |
| Vincristine + methotrexate + *cis*-platinum | 3 | 7‡ | 6 |

* >50% fall in hCG concentration.

† >10-fold fall in hCG concentration.

‡ 1 patient received both treatments in sequence.

patients are in complete remission and off treatment (range 10–25 months).

In summary, 17 patients with drug-resistant gestational choriocarcinoma were treated with high-dose (120 mg/m$^2$) *cis*-platinum. No activity was seen in 2 patients with high-dose *cis*-platinum alone, but in combination with vincristine and methotrexate there were 6 responses and 7 improvements, whilst only 3 patients had progressive disease. *cis*-Platinum used in this combination is clearly active, and requires further assessment in drug-resistant gestational choriocarcinoma.

We would like to thank the National Cancer Institute, Bethesda, U.S.A., for supplying us with *cis*-platinum for this study.

### REFERENCES

AMIEL, J. L., DROZ, J. P. & TURSZ, T. (1978) Tumeurs placentaires resistantes aux chimiothérapies habituelles: Traitement par le *cis*-diamine dichloroplatinum (2 cases). *Nouv. Presse Med.*, **7**, 1933.

BAGSHAWE, K. D. (1976) Risk and prognostic factors in trophoblastic neoplasia. *Cancer*, **38**, 1373.

BAGSHAWE, K. D. (1977) Treatment of trophoblastic tumours. *Recent Results Cancer Res.*, **62**, 192.

HAYES, D. M., CVITKOVIC, E., GOLBEY, R. B., SCHEINER, E., HELSON, L. & KRAKOFF, R. H. (1977) High dose *cis*-platinum diammine dichloride. Amelioration of renal toxicity by mannitol diuresis. *Cancer*, **39**, 1372.

KARDANA, A. & BAGSHAWE, K. D. (1976) A rapid, sensitive and specific radioimmunoassay for human chorionic gonadotrophin. *J. Immunol. Methods*, **9**, 297.

NEWLANDS, E. S. (1978) Preliminary experience with high dose *cis*-platinum and the epipodophyllin derivative, VP16-213, in resistant malignant teratomas and choriocarcinomas. *Current Chemother.*, **2**, 1315.

PRESTAYKO, A. W., D'AOUST, J. C., ISSELL, B. F. & CROOKE, S. T. (1979) Cisplatin (*cis*-diamminedichloroplatinum II). *Cancer Treat. Rev.*, **6**, 17.

ROZENCWEIG, M., VON HOFF, D. D., SLAVIK, M. & MUGGIA, F. M. (1977) *cis*-diamminedichloroplatinum II. A new anti-cancer drug. *Ann. Int. Med.*, **86**, 803.